# Supplementary material for: Strengthening government’s response to COVID-19 in Indonesia: A modified Delphi study of medical and health academics
Source: PLoS One. 2022 Sep 29;17(9):e0275153. doi: 10.1371/journal.pone.0275153 (PMC9522309; doi:10.1371/journal.pone.0275153)
Supplement: Attachment — Submitted filename: Responses to reviewers PLOS ONE v1.docx [file pone.0275153.s004.docx]

*Dear Editor, many thanks for the comments. Please find below point-by-point responses to the comments.*

1. Please ensure that your manuscript meets PLOS ONE's style requirements, including those for file naming. The PLOS ONE style templates can be found at

https://journals.plos.org/plosone/s/file?id=wjVg/PLOSOne_formatting_sample_main_body.pdf and

https://journals.plos.org/plosone/s/file?id=ba62/PLOSOne_formatting_sample_title_authors_affiliations.pdf

*We have reviewed the style requirements and adjusted the manuscript accordingly.*

2. Please provide additional details regarding participant consent. In the ethics statement in the Methods and online submission information, please ensure that you have specified what type you obtained (for instance, written or verbal, and if verbal, how it was documented and witnessed). If your study included minors, state whether you obtained consent from parents or guardians. If the need for consent was waived by the ethics committee, please include this information.

*No minors were involved in our study, and we obtained written informed consent from all participants. We've added the following text to the ethic sub-section of the method section:*

*"Written informed consent was required before a participant could start filling the online questionnaire."*

3. In the Methods section, please describe in detail by what method the webinar discussion sessions were conducted. For instance, please provide information on the duration of the webinar, who conducted the webinar and their training experience and background, duration and finally how content was recorded and analysed.

*We have added the following text in the method section:*

*"**The webinar was delivered via Zoom, facilitated by the authors and completed in 1,5 hours. During the webinar, respondents were asked whether they agree with the presented results and priority ranking. The authors documented their responses and the entire webinar was audio and video recorded."*

Reviewer #1: The authors aim to evaluate and provide further direction to Indonesia's Covid-19 response. To do so, they rely on a Delphi-style survey using the COVID-SCORE tool. Ultimately, this allows them to evaluate the government's response and identify key areas for improvement. This is clearly an important topic that merits consideration. Sadly, in the current form, I cannot recommend publication. I will not comment on individual aspects of the manuscript but rather I wish to focus on what I see as the two related major hindrances: the methodology and the discussion/recommendations.

*Thank you for highlighting the importance of the topic. We address below the two hindrances noted by the reviewer*

The methodology is very rigid and largely seems to be a the same questionnaire applied twice. Though there are optional short responses, there seems to be little room to obtain more in-depth observations and recommendations from the experts.

*We concur that there are limitations to the Delphi technique. Notwithstanding, this technique is a well-established approach to answering a research question by identifying a consensus view across subject experts. The Delphi technique has been used internationally to investigate a wide variety of issues, including health issues. This technique is primarily used in health sciences by researchers when the available knowledge is incomplete or subject to uncertainty, and other methods that provide higher levels of evidence cannot be used. Thus, we believe applying the Delphi method rigorously can provide answers which are useful and credible. We have added the following text in the method section to highlight this*

*"* *The Delphi technique is a well-established approach to answering a research question by identifying a consensus view across subject experts [11]. This technique is commonly used in developing consensual guidance on best practice and exploration of a field beyond existing knowledge and the current conceptual framework [12]. The Delphi technique has been used internationally to investigate a wide variety of issues, including health issues. This technique is primarily used in health sciences by researchers when the available knowledge is incomplete or subject to uncertainty, and other methods that provide higher levels of evidence cannot be used [13]."*

An open-ended survey or focus group discussion with selected experts would be needed to do so. Though you mention having held a webinar, it is not clear if this helped build on the results in any way.

*We agree that a follow-up survey and FGDs would be useful. These have been carried out subsequently and has led to a separate manuscript which has been published elsewhere. We have added the following text in the result section to clarify this:*

*"* *During the webinar to discuss these results, experts confirmed their agreement with the presented results and priority ranking. They also suggested recommendations for follow-ups of the study. Accordingly, the results were shared with the National Planning Development Agency and informed the development of a follow-up study. Some of the results of this subsequent study has now been published elsewhere [16]."*

This leads to the second major issue. The recommendations in the manuscript are vague and therefore of limited use. For example, you mention the importance of a testing strategy or contact tracing. Yet there is little specific insight or recommendations on how these items are being specifically implemented in Indonesia and how they can be improved. In other words, the manuscript merely emphasizes the importance of such items, but there is little country-specific information provided. Moving forward, more specific recommendations and context need to be provided. This can be done by sourcing additional local news/policy/literature as well as by conducting a third qualitative step in your Delphi study, whereby you validate and discuss the results in one or more focus groups with relevant experts. I feel such a step is needed to deepen the manuscript and maximize its relevance.

*We have added the following texts in the discussion section to provide additional country specific context as requested:*

*"* *The only source of official information at the early phase of the Pandemic in Indonesia came from the presidential office and the Indonesian Health Ministry. The government did not appear to have a "sense of crisis" even as of early February 2020. This was exacerbated by "antiscience" statements issued by government officials [19]. The government appointed a spokesman who informed the progress of the spread of COVID-19 through one door regularly and periodically twice a day. However, when the number of cases increased in several areas, mayors and governors also issued statements, which often contradict the official data held by the presidential office and Minister of Health [20]."*

*"The Indonesian government has implemented government-centered public communication without prohibiting media, including social media, from spreading general information about COVID-19 [25]. Thus, the primary sources of information for the public during the pandemic are social media, online media, and mass media. The most popular sources for preventive measures are social media (83.6%), television (78.5%), and WhatsApp (76.0%) [26]. Notably, Indonesia faces problems with the spread of misleading and harmful messages that hamper COVID-19 prevention efforts. The Ministry of Communications and Informatics monitors the spread of COVID-19 related mis/dis-information daily, with records of 1,550 hoaxes related to Covid-19 dan 177 hoaxes related to Covid-19 vaccines from January 2020 to April 2021 [27]. In response, the government has partnered with digital technology-based industries and social media influencers for disseminating information [28]."*

*"The gap between the RT-PCR testing capacity in laboratories and the number of suspected cases to be tested remains significant. Accordingly, the government adopted the WHO recommendation to use antigen detection rapid diagnostic test (Ag-RDT) to diagnose SARS-CoV-2 infection in settings where RT-PCR is unavailable or where prolonged turnaround precludes clinical utility [33]. The utilization of Ag-RDT has augmented the country's testing capacity. More recently, the government has also adopted GeNose C19, a breath-based COVID-19 detection device developed by Indonesian scientists. Initial trial on the device revealed 89-92% sensitivity for detecting positive cases, comparable to RT-PCR (89%) and antigen-based rapid test (89.9%) [34]. The tool that detects the infection through exhaled volatile organic compounds gives results in only three minutes. It costs only US$ 0.7-1.7 or around 11-140 times cheaper than the other two tests. [34] The Ministry of Transportation has deployed the tool in train stations and bus terminals, hoping that the device can speed up the passengers screening process."*

*"The Ministry of Information and Communication launched a mobile application called PeduliLindungi. The app enables users to compile data related to the spread of COVID-19 in their communities to help bolster the Indonesian government's efforts to trace and track confirmed cases. Users are expected to register as participants and share their locations when travelling and trace whether they have contacted persons exposed to COVID-19. The app will also alert users entering crowds or COVID-19 red zones, namely areas with confirmed COVID-19 cases [39]."*

*We have also added the following text to the conclusion section*

*"To revamp the COVID-19 response in Indonesia, the government first must urgently need to strengthen capacity to ensure consistent, credible and targeted communication while adopting a more inclusive and empathic communication style to address public concerns. Second, the Indonesian government need to ensure universal access to reliable COVID-19 test by expanding lab infrastructure and to ensure universal access to reliable COVID-19 test by expanding lab infrastructure, facilitating operational readiness, and scaling up implementation of proven alternative/complementary tests to RT-PCR. Finally, the government need to boost contact tracing implementation capacity and facilitate contact tracing for all positive cases, involving key stakeholders in further development of the existing contact tracing system (i.e. PeduliLindungi) as well as its evaluation and quality assurance."*

Reviewer #2: The paper is important to inform the Indonesia government in tackling the COVID-19 Pandemic. It is well written in very good English and understandable to read. The strength of this paper is the implementation of the COVID-19 assessment scorecard (COVID-SCORE) in a modified Delphi method. The results may be used to develop new models of preparedness and response for future public-health threads.

To make it better, the authors may:

• Line 156  please clarify that the second cycle is the final results (no other returning the questionnaire to the researchers again)

*We have revised the sentence as follow to emphasize that the second cycle was the final cycle:*

*"The result of the second (final) cycle was analyzed, and feedback to all participants a week later."*

• Line 189  it may be better if the majority comes first (68.5% doctoral degree vs 31.5% master degree)

*We have revised as follow in line with the suggestion:*

*"Most of the participants hold a doctoral degree (48, 68.5%) and the rest hold a master degree (22, 31.5%)."*

• Line 192-193  how about the 17 participants who directly involved with COVID-19 response team? Why were they not excluded from the survey related to their possibility of subjectivity?

*The 17 participants who were directly involved with the COVID-19 response were not excluded as we consider their involvement to be added value in providing insights as those who were close with the realities of COVID-19 response in the field.*

• Line 207/ Table 1. There is a column of "Neither agree nor disagree" and the columns for assessment of comments consisting of positive, negative, or mixed comments. However, in the analysis, only 'agreement' or 'disagreement' are described. Also, there are no definitions for positive, negative, and mixed sentiment.

*We have now deleted the positive, negative or mixed comments as the focus of our analysis is on the agreement/disagreement and priority ranking*

• Line 468. After stating the Wuhan government failure in early communication, it will be more informative if there are any data about communication of Indonesia government in the early Pandemic, such as the early response of the previous Minister of Health who was not able to build the public trusts. Also, the role of MoH spokesman should be discussed.

We have added the following text to the discussion section as advised:

*"The only source of official information at the early phase of the Pandemic in Indonesia came from the presidential office and the Indonesian Health Ministry. The government did not appear to have a "sense of crisis" even as of early February 2020. This was exacerbated by "antiscience" statements issued by government officials [19]. The government appointed a spokesman who informed the progress of the spread of COVID-19 through one door regularly and periodically twice a day. However, when the number of cases increased in several areas, mayors and governors also issued statements, which often contradict the official data held by the presidential office and Minister of Health [20]."*

• Line 487-488. The important of social media in communication  is there any data about the quality of government's social media and how the government tackle the false pandemic information. There are also a few independent organizations that manage social media account to provide pandemic information and also to identify discrepancy data in COVID-19 daily report (e.g., @pandemictalks; @kawalcovid19)  it would be interesting to discuss their role in public health communication and how the government response.

*"The Indonesian government has implemented government-centered public communication without prohibiting media, including social media, from spreading general information about COVID-19 [25]. Thus, the primary sources of information for the public during the pandemic are social media, online media, and mass media. The most popular sources for preventive measures are social media (83.6%), television (78.5%), and WhatsApp (76.0%) [26]. Notably, Indonesia faces problems with the spread of misleading and harmful messages that hamper COVID-19 prevention efforts. The Ministry of Communications and Informatics monitors the spread of COVID-19 related mis/dis-information daily, with records of 1,550 hoaxes related to Covid-19 dan 177 hoaxes related to Covid-19 vaccines from January 2020 to April 2021 [27]. In response, the government has partnered with digital technology-based industries and social media influencers for disseminating information [28]."*

• Line 494-510 about enhancing testing and contact tracing  it would be useful if the authors update the impacts of latest diagnostic methods used in Indonesia (e.g., RDT antigen and Genose) as these methods have already been regulated by the government to trace and screen."

*We have added the following text to the discussion section as advised:*

*"The gap between the RT-PCR testing capacity in laboratories and the number of suspected cases to be tested remains significant. Accordingly, the government adopted the WHO recommendation to use antigen detection rapid diagnostic test (Ag-RDT) to diagnose SARS-CoV-2 infection in settings where RT-PCR is unavailable or where prolonged turnaround precludes clinical utility [33]. The utilization of Ag-RDT has augmented the country's testing capacity. More recently, the government has also adopted GeNose C19, a breath-based COVID-19 detection device developed by Indonesian scientists. Initial trial on the device revealed 89-92% sensitivity for detecting positive cases, comparable to RT-PCR (89%) and antigen-based rapid test (89.9%) [34]. The tool that detects the infection through exhaled volatile organic compounds gives results in only three minutes. It costs only US$ 0.7-1.7 or around 11-140 times cheaper than the other two tests. [34] The Ministry of Transportation has deployed the tool in train stations and bus terminals, hoping that the device can speed up the passengers screening process."*

• P 522-523, about app-based digital tracing  there is an interesting article to enrich the discussion: Gasser U, Ienca M, Scheibner J, Sleigh J, Vayena E. Digital tools against COVID-19: taxonomy, ethical challenges, and navigation aid. Lancet Digit Health. 2020 Aug;2(8):e425-e434. doi: 10.1016/S2589-7500(20)30137-0. Epub 2020 Jun 29. PMID: 32835200; PMCID: PMC7324107.

*We have incorporated the recommended reference into the discussion section as well:*

*"However, these digital tools still need to be ensured to be scientifically and ethically sound to ensure widespread public trust and uptake [40]. Typological analysis and established frameworks in public health and big data ethics can aid governments and other actors in discerning the complex ethical–legal landscape in which these digital tools will operate [40]. "*

• Related to app-based digital tracing, kindly update the Indonesian government-sponsored tracing app, PeduliLindungi. PeduliLindungi is an android-based mobile application developed by the Indonesian government to mitigate the spread of COVID-19. The application relies on the willingness of people to share their current health condition and make it available for tracking using global positioning system (GPS) and Bluetooth technology.

*We have added the following text to the discussion section as advised:*

*"The Ministry of Information and Communication launched a mobile application called PeduliLindungi. The app enables users to compile data related to the spread of COVID-19 in their communities to help bolster the Indonesian government's efforts to trace and track confirmed cases. Users are expected to register as participants and share their locations when travelling and trace whether they have contacted persons exposed to COVID-19. The app will also alert users entering crowds or COVID-19 red zones, namely areas with confirmed COVID-19 cases [39]. "*

• In the analysis section, the authors mentioned the webinar between experts and the National Planning Development Agency to discuss findings of the study it would be nice if authors discuss the responses from the National Planning Development Agency during the webinar and provide the outcomes of the meeting.

*We have added the following text to the result section as advised:*

*"**During the webinar to discuss these results, experts confirmed their agreement with the presented results and priority ranking. They also suggested recommendations for follow-ups of the study. Accordingly, the results were shared with the National Planning Development Agency and informed the development of a follow-up study. Some of the results of this subsequent study has now been published elsewhere [16]."*
