# Supplementary material for: Strengthening government’s response to COVID-19 in Indonesia: A modified Delphi study of medical and health academics
Source: PLoS One. 2022 Sep 29;17(9):e0275153. doi: 10.1371/journal.pone.0275153 (PMC9522309; doi:10.1371/journal.pone.0275153)
Supplement: Attachment — Submitted filename: Reviewer 2_Last minor revision.docx [file pone.0275153.s005.docx]

Thank you for addressing my issues. I would like to congratulate you on the improvement in your paper. I only have one issue left regarding the GeNose C19 since there was updating news.

You added:

“More recently, the government has also adopted GeNose C19, a breath-based COVID-19 detection device developed by Indonesian scientists. Initial trial on the device revealed 89-92% sensitivity for detecting positive cases, comparable to RT-PCR (89%) and antigen-based rapid test (89.9%) [34]. The tool that detects the infection through exhaled volatile organic compounds gives results in only three minutes. It costs only US$ 0.7-1.7 or around 11-140 times cheaper than the other two tests. [34] The Ministry of Transportation has deployed the tool in train stations and bus terminals, hoping that the device can speed up the passengers screening process."

As we know that the GeNose C19 is now halted by the Government upon its controversy. I would like you to delete the discussion about the GeNose C19 to avoid the confusion. Kindly highlight the effort from the Government in providing effective and reliable COVID-19 screening modalities, and also how the Government take the expert and epidemiologist recommendation. Thank you.
