# Supplementary material for: Strengthening government’s response to COVID-19 in Indonesia: A modified Delphi study of medical and health academics
Source: PLoS One. 2022 Sep 29;17(9):e0275153. doi: 10.1371/journal.pone.0275153 (PMC9522309; doi:10.1371/journal.pone.0275153)
Supplement: Attachment — Submitted filename: Response to Reviewers PLOS One.docx [file pone.0275153.s006.docx]

Dear Editor,

Thank you for the reviewers' comments. We've now revised the manuscript in line with the comments. Please find below point-by-point responses to the comments.

Best regards,

Yodi Mahendradhata

---------------------------------------------------------------

*Reviewer #2: Thank you for addressing my issues. I would like to congratulate you on the improvement in your paper. I only have one issue left regarding the GeNose C19 since there was updating news.*

*You added:*

*"More recently, the government has also adopted GeNose C19, a breath-based COVID-19 detection device developed by Indonesian scientists. Initial trial on the device revealed 89-92% sensitivity for detecting positive cases, comparable to RT-PCR (89%) and antigen-based rapid test (89.9%) [34]. The tool that detects the infection through exhaled volatile organic compounds gives results in only three minutes. It costs only US$ 0.7-1.7 or around 11-140 times cheaper than the other two tests. [34] The Ministry of Transportation has deployed the tool in train stations and bus terminals, hoping that the device can speed up the passengers screening process."*

*As we know that the GeNose C19 is now halted by the Government upon its controversy. I would like you to delete the discussion about the GeNose C19 to avoid the confusion. Kindly highlight the effort from the Government in providing effective and reliable COVID-19 screening modalities, and also how the Government take the expert and epidemiologist recommendation. Thank you.*

The text on GeNose C19 in the discussion section has been deleted and replaced with text to highlight government effort in reaching WHO recommended testing rate and enhancing capacity for detecting variants of concerns as follow:

" As of September 19 2021, all provinces in Indonesia have managed to meet the WHO recommended testing rate of at least one person per 1000 population per week [34]. Additionally, to enhance capacity to detect variants of concerns, the Ministry of Health has incorporated 21 institutions into its SARS-CoV-2 genome surveillance laboratory network, with the capacity of around 1,750 – 2,700 tests per month. As of August 29 2021, Indonesia has shared 5,755 (0.142% cases) sequences since the beginning of the pandemic, according to GISAID (Global Initiative on sharing all Influenza data). By August 2021, the Ministry of Health announced that the Delta Variant had been detected in six provinces, i.e. DKI Jakarta, West Java, East Kalimantan, Central Java, East Nusa Tenggara, and Bali 1 2021 [35]."

*Reviewer #3: Research Summary and overall impression*

*The study assessed the response efforts by the Indonesian government to the COVID-19 pandemic from the time of first outbreak in February, 2020 in Indonesia. This work involved medical and health academics from ten universities across Indonesia, whose views on 20 prepared statements were captured using a five-point Likert scale in a modified Delphi study. Suggested areas requiring improvement were also put forth by participants.*

*Overall, this was a good study, which has been well-presented. This is a relevant study as far as COVID-19-related Public Health issues are concerned. Some insight is provided the reader into the strengths and shortfalls of the Indonesian government in its handling of the COVID-19 outbreak in the archipelagos – issues which are common to many countries across the globe, developed and developing alike.*

*There however are a few observations which the authors should note:*

*Major Issues*

*1. Line 369: (majority agreement, 51.4% disagree) – this is a rather confusing presentation. Although one deduces from reading further that the first agreement is actually degree of consensus, and the second agreement with percentage indicates the kind of agreement (whether agreed or disagreed), it could adversely impact the smooth reading and flow of the manuscript.*

*I would suggest that different categorizations (such as 'poor consensus', 'consensus', 'good consensus' and 'strong consensus') could be adopted instead of what is currently being used (i.e. 'no consensus', 'majority agreement', 'consensus', and 'strong consensus'). This could also greatly improve understanding at first read. Same issue applies to Table 1 as well*

We concur that the term "majority agreement" may lead to confusion. Thus, the categorization has now been modified to "no consensus", "low consensus level", "moderate consensus level" and "high consensus level." This modification follows categorization described by Meskell et al.^[1]^ The modified categorization has been described in the method section as follow:

"High consensus level" was defined as ≥95% agreement in the rating of the single statements by the panellists; "Moderate consensus level" was defined as the agreement of 75–94%, "Low consensus level" was defined as the agreement of 50-74%, and "No consensus" was recorded if the agreement was <50%."

The new categorization has been applied to Table one and the relevant texts in the result dan discussion sections.

*2. Table 1: I'm not so sure what the relevance of the p-value column is in this work. No mention is made of it in either the results or the discussion sections. Meanwhile, this could serve to justify why it was needful to conduct the study twice with the same participants. I think it would enrich the manuscript if they could insert such information*

The following additional texts have now been inserted into the method section:

" In the second cycle, respondents had the opportunity to revise their answers. We calculated p-values to assess the significance of the change in the degree of consensus for each statement between the cycles."

And the result section:

" Compared to the first cycle, the consensus degree was improved for 15 statements and reduced for four statements. However, a significant change (p < 0.05) of consensus degree between the cycles was only observed for one statement ("Mental health outreach services have been expanded to meet increased demand")."

*Minor Issues*

*1. Lines 439 – 440: I think the authors should be more careful with their tenses. It would be helpful if they could go through the entire manuscript to correct these issues.*

The noted text has been revised as follow, and the rest of the manuscript has been thoroughly checked using Grammarly Business :

" The government have released financial incentives for the poor and encouraged older adults and young children to stay at home."

*2. Lines 389 – 392: Unclear statement. Please revise.*

The noted text has been revised as follow:

" Respondents suggest campaigns should adapt local wisdom and use language that should be easy for people with low literacy to understand. The use of the internet and social media to share information has discriminated population living in rural without access to the internet or television."

*3. Lines 518 - 519: The clause 'through social media and employ strategies for improved communication management to citizens through social media…' was repeated. Please revise.*

The noted text has been revised as follow:

" The Indonesian government thus need to improve their efforts to disseminate information on the pandemic and employ strategies for improved communication management to citizens through social media and mainstream information sources [24]."

*Other/General remarks:*

*I think the study was well-done and well-presented. However, attention must be paid to modify the categorizations; as well as correct tenses and other minor grammatical errors. This would greatly enhance the readability of the study.*

*I would recommend it for publication. Thank you.*

The categorization has now been modified as described above, and the manuscript has now been rechecked for grammatical errors using Grammarly Business.

1. Meskell P, Murphy K, Shaw D, Casey D. Insights into the use and complexities of the Policy Delphi technique. Nurse Researcher. 2014; 21:3, 32-39.
