# Supplementary material for: Strengthening government’s response to COVID-19 in Indonesia: A modified Delphi study of medical and health academics
Source: PLoS One. 2022 Sep 29;17(9):e0275153. doi: 10.1371/journal.pone.0275153 (PMC9522309; doi:10.1371/journal.pone.0275153)
Supplement: Attachment — Submitted filename: Review report 1.1.docx [file pone.0275153.s007.docx]

**Comments to the Author**

Thanks for the authors for introducing us how medical and health academics view the Indonesian government's handling of the COVID-19 and which areas of health systems need to be prioritized. However, there are several concerns:

1. Although the author explains the reasons for using the Delphi method in page 5 lines 93-95, this kind of research does not require the Delphi method (time-consuming and laborious). The same result would have been obtained if a questionnaire had been used. The commonly used Delphi method is only used to forecast, put forward the index system and determine the specific index.
2. In the research design section, *the COVID-19 assessment scorecard* consists of 19 policy statements [1], but the author noted 20 in the manuscript.
3. It is odd for the authors to use the Cambridge Dictionary's definition of scholarship to select a study population. The views of the health sector and healthcare providers on policies to control COVID-19 are even more important. The study was underrepresented.
4. In the results section, it is more intuitive to use the expert status table. For example, age can be expressed in three groups.
5. Please explain in the methods section why the P value is calculated. In general, the Delphi method does not require the calculation of P- values.
6. It is too tedious to present all policy statements in the results. Simplify them.
7. From the results of this study, moderate consensus was reached on only 5 statements, low consensus was reached on 13 statements, and no consensus was reached on 2 statements. In the Delphi method, the reliability of the results should be proved. However, the expert' authority coefficient (Cr) and the coefficient of variation (CV) are necessary. The reliability and representativeness of the manuscript are worrisome.

[1] Lazarus JV, Binagwaho A, El-Mohandes AAE, Fielding JE, Larson HJ, Plasència A, et al. Keeping governments accountable: the COVID-19 Assessment Scorecard (COVID- SCORE). Nat Med. 2020 Jul;26(7):1005-1008.
