# Supplementary material for: Strengthening government’s response to COVID-19 in Indonesia: A modified Delphi study of medical and health academics
Source: PLoS One. 2022 Sep 29;17(9):e0275153. doi: 10.1371/journal.pone.0275153 (PMC9522309; doi:10.1371/journal.pone.0275153)
Supplement: Attachment — Submitted filename: PLOS One response to reviewers YM v1.docx [file pone.0275153.s008.docx]

Dear Editor,

Thank you for the reviewers’ comments. Please find below our point-by-point responses.

Best regards,

Yodi Mahendradhata

----------------------------------------------------------------------------

Reviewer #3: Overall Impressions:

All major issues have been catered for by the co-authors. There are however a number of minor issues, mainly typographical and grammatical, that the co-authors would need to address:

Issues:

Location Issue Line 19 ‘government’s’ was repeated twice

*This has been corrected as follow:*

*“We aimed to investigate how medical and health academics view Indonesian government's handling of COVID-19 and which areas of health systems need to be prioritized to improve the government's response to COVID-19.”*

Line 19 Remove ‘the’ in ‘…government’s handling of the COVID-19…’

*This has been corrected as follow:*

*“We aimed to investigate how medical and health academics view Indonesian government's handling of COVID-19 and which areas of health systems need to be prioritized to improve the government's response to COVID-19.”*

Line 25 Remove ‘have’ in the statement: ‘All participants who have completed…’

*This has been corrected a follow:*

*“All participants who completed the first cycle were invited to participate in the second cycle.”*

Line 27 ‘rank’ should be ‘ranked’

*This has been corrected as follow:*

*“They had the opportunity to revise their answers based on the previous cycle's results and ranked a list of actions to improve government response.”*

Replace ‘priority with ‘list’

*This has been corrected as follow:*

*“They had the opportunity to revise their answers based on the previous cycle's results and ranked a list of actions to improve government response.”*

Line 31 Replace ‘strengthen’ with ‘strengthening’

*This has been corrected as follow:*

*“strengthening capacity to ensure consistent, credible and targeted communication while adopting a more inclusive and empathic communication style to address public concerns”*

Line 33 Replace ‘ensure’ with ‘ensuring’

*This has been corrected as follow:*

*“ensuring universal access to reliable COVID-19 test by expanding lab infrastructure, facilitating operational readiness, and scaling up implementation of proven alternative/complementary tests to RT-PCR;”*

Line 35 Replace ‘boost’ with ‘boosting’

*This has been corrected as follow:*

*“boosting contact tracing implementation capacity and facilitating contact tracing for all positive cases, involving key stakeholders in further development of the existing contact tracing system (i.e. PeduliLindungi) as well as its evaluation and quality assurance.”*

Line 36 Replace ‘facilitate’ with ‘facilitating’

*This has been corrected as follow:*

*“boosting contact tracing implementation capacity and facilitating contact tracing for all positive cases, involving key stakeholders in further development of the existing contact tracing system (i.e. PeduliLindungi) as well as its evaluation and quality assurance.”*

Line 122 Replace ‘has’ with ‘had’

*This has been corrected as follow:*

*“To be considered a participant in this study, each candidate had to fulfil the following criteria”*

Line 132 Replace ‘university’ with ‘respective universities’

*This has been corrected as follow:*

*“To ensure that respondents have the capacity and capability to participate in the survey, we then performed snowball sampling by asking senior lecturers to recommend a list of academics from their respective universities.”*

Line 176 Replace ‘will be’ with ‘was’

*This has been corrected as follow:*

“For the ranking score, each priority action was be scored according to its sum of rank.”

Line 177 Replace ‘will be’ with ‘was considered’

*This has been corrected as follow:*

*The lowest total score was considered the highest priority.*

Line 188 Replace ‘can’ with ‘could’

*This has been corrected as follow:*

*“Therefore, they could freely submit their ideas, unbiased by the identities or pressures of others.”*

Line 190 Replace ‘are’ with ‘were’

*This has been corrected as follow:*

*“All participants were coded in the analysis; thus, their opinions and comments were anonymous to the investigators.”*

Line 225 Replace ‘has’ with ‘have’

*This has been corrected as follow:*

*“Some of the results of this subsequent study have now been published elsewhere”*

Line 289 Capitalize ‘low’

*This has been corrected as follow:*

*“Low consensus level was achieved for the following statements:…”*

Lines 290, 292, 298, 300, 301, 302, 304, 305 Replace ‘,’ with ‘;’

*This has been corrected as follow:*

*“….the government has tried to maintain robust epidemiological databases at national and local levels (71.4% agree); the government should involve in country private manufacturers to produce critical equipment rapidly as part of corporate social responsibility (67.1% agree); a pandemic preparedness team that includes public health and medical experts is coordinating the national response (55.7% agree); infection prevention and care guidelines and protocols are comprehensive and up to date (61.4% agree); task sharing, task shifting and telehealth are being used to optimize the delivery of health care services (52.9% agree); public health measures have been implemented to protect people in institutions and other confined settings (55.0% agree); the government has target the entire population (68.5% disagree), there were enough qualified healthcare workers and medical equipment to meet national needs (67.1% disagree); the government has communicated clearly and consistently about COVID-19 (51.4% disagree);* *the government has enough funding and infrastructure to care for all COVID-19 patients, particularly in the long run (64.3% disagree); public health experts, government officials, and academic researchers agree on COVID-19 nomenclature and have clearly explain the reason for public health measures (54.2% disagree); access to regular health services was uninterrupted (64.3% disagree); and that appropriate measures have been taken to protect members of vulnerable groups, such as the elderly, the poor, migrants, and the homeless (61.4% disagree).”*

Line 300 Replace ‘communicate’ with ‘communicated’

*This has been corrected as follow:*

*“the government has communicated clearly and consistently about COVID-19 (51.4% disagree);”*

Line 301 Replace ‘have’ with ‘has’

*This has been corrected as follow:*

*“the government has enough funding and infrastructure to care for all COVID-19 patients, particularly in the long run (64.3% disagree)”*

Line 303 Replace ‘has’ with ‘have’

*This has been corrected as follow:*

*“public health experts, government officials, and academic researchers agree on COVID-19 nomenclature and have clearly explain the reason for public health measures (54.2% disagree);”*

Line 310 Replace ‘eight’ with ‘eighth’

*This has been corrected as follow:*

*“It ranked eighth on priority recommended actions.”*

Line 337 Modify ‘at what extent does’ to ‘the extent to which’

*This has been corrected as follow:*

*“Thus, the extent to which public health experts have been involved in the response team remains questionable.”*

Line 359 Replace ‘needs’ with ‘needed’

*This has been corrected as follow:*

*“Payment for telehealth services was also another issue that needed to be discussed by policymakers.”*

Line 394 Replace ‘suggest’ with ‘suggested that’

*This has been corrected as follow:*

*“Respondents suggested that campaigns should adapt local wisdom and use language that should be easy for people with low literacy to understand.”*

Line 396 Modify ‘rural’ to read ‘rural areas’

*This has been corrected as follow:*

*“The use of the internet and social media to share information has discriminated population living in rural areas without access to the internet or television.”*

Line 410 Replace ‘issue’ with ‘issues’

*This has been corrected as follow:*

*“There were also issues regarding terms used for infection control, disease transmission and treatment.”*

Line 417 Modify ‘price skyrocketing’ to read ‘prices were skyrocketing’

*This has been corrected as follow:*

*“Medical personal protective equipment was scarce in every part of the country, and prices were skyrocketing after months of COVID-19.”*

Line 446 Replace ‘philanthropic’ with ‘philanthropies’

*This has been corrected as follow:*

*“Many non-governmental organizations and philanthropies raise funds to support those affected by the COVID-19 pandemic, including the poor, elderly, and frontline healthcare workers.”*

Line 504 Replace ‘contradict’ with ‘contradicted

*This has been corrected as follow:*

*“However, when the number of cases increased in several areas, mayors and governors also issued statements, which often contradicted the official data held by the presidential office and Minister of Health”*

Line 531 Replace ‘dan’ with ‘and’

*This has been corrected as follow:*

*“The Ministry of Communications and Informatics monitors the spread of COVID-19 related mis/dis-information daily, with records of 1,550 hoaxes related to Covid-19 and 177 hoaxes related to Covid-19 vaccines from January 2020 to April 2021”*

Reviewer #4: Line 15: Please make sure to use one common tense (either present or past) throughout the abstract. There are multiple grammatical mistakes in this paragraph. Please correct them.

*The abstract has been thoroughly checked with Grammarly Premium and corrected accordingly*

Line: 19: Please omit the repeated words.

*This has been corrected as follow:*

Line 23: Please clarify how many rounds of data collection was performed.

*The sentence has been revised as follow:*

*“We invited medical and health academics from ten universities across Indonesia to take part in the two-round Delphi study”*

Line 46: Please be consistent when characterizing the COVID-19 as a pandemic. Here the authors have depicted it as an epidemic, which may confuse the readers.

*The sentence has now been revised as follow:*

*“Governments' failures to suppress the epidemic pandemic is disappointing and costly.”*

Line 60: Please explain the term "zero case" - is it an academic term? If so, please provide a reference for it - otherwise please explain clearly for the readers.

*The sentence has now been revised as follow:*

*“The initial claim of no case reported by Indonesia, before the first two confirmed cases, was questioned by many”*

Line 62: Should it be specific quarantines, or specific policies/instructions/guidelines for quarantines? Please clarify.

*The sentence has now been revised as follow:*

*“During this period, the government did not issue travel restrictions and specific policies for quarantines of travellers coming in/coming back to Indonesia, despite reports of increasing COVID-19 cases in neighbouring countries”*

Line 64: Is there any data regarding Indonesian nationals versus tourists arriving in the country during that period of time? Please provide references as Indonesia is a known tourist country.

*We could not find the requested data*

Line 67: Please provide information on the first COVID-19 related death occurring in Indonesia - when did it occur and how did that impact the governments' decisions?

*The sentence has now been revised as follow:*

*“After the initial and subsequent reports of infections as well as the first death reported from coronavirus on March 11, 2020, the Indonesian government realized the seriousness of the situation [8, 9].”*

Line 75: When the authors mention "Indonesia's vulnerable health system" - what do they mean? Please provide some context as to why the healthcare system is vulnerable in Indonesia.

The sentence has now been revised as follow:

*“The impact on Indonesia's vulnerable health system, due to insufficient health workforce and health care infrastructure, will be devastating if COVID-19 continues the trajectory observed in other countries.”*

Line 76: Please clarify what the non-COVID-19 related impacts are.

*This has now been revised as follow:*

*“Meanwhile, the economic, social, and non-COVID-19 related health system impact (e.g. disruptions of non-COVID-19 related health services) has already taken a significant toll on Indonesia.”*

Line 78: Please be consistent with WHO announcements on COVID-19 being a pandemic.

The sentence has now been revised as follow:

*“Coordinated and comprehensive actions to suppress the COVID-19 epidemic pandemic in Indonesia need to be enhanced.”*

Line 82: Please clarify the difference between health professionals (line 80) and health academics (line 82). Please use one term consistently throughout the paper.

*The sentences have been revised as follow:*

*“This study aims to consolidate advice from medical and health academics for the government to enhance the COVID-19 response in Indonesia. The study objectives are twofold. First, investigate how medical and health academics view the government of Indonesia's handling of the COVID-19 epidemic.”*

Line 91: A better term that can be used here is "to achieve the study objectives".

*The sentence has now been revised as follow:*

*“We conducted a modified Delphi study to address achieve the study objectives”*

Line 96: Please correct for double spaces throughout the manuscript. Please use single spaces.

*In accordance to PLOS One guideline, we retain the double-spaced manuscript text.*

Line 102: If possible, please provide references of other studies that have used a similar methodology to study the COVID-19 pandemic.

*We have not found other studies using the same method for the COVID-19 pandemic*

Line 104: Please clarify what the "modified Delphi study" is. Is this different from the original method? If so, how and why is it different? Please clarify for the readers.

*We’ve inserted the following additional sentence for clarity as requested:*

*“The modified Delphi method we used is similar to the standard Delphi method in terms of procedure (i.e., a series of rounds with selected experts) and intent (i.e., to arrive at consensus). The major modification consists of beginning the process with a set of carefully selected items.”*

Line 114: Please provide some context as to what type of "adopted statements" were utilized in the study. Are these statements from the Indonesian government? If so, which government body issued them and in what context? Was there a specific time frame from which the statements were collected? What was the inclusion criteria for these statements? Please clarify.

*The sentence has now been revised as follow:*

*“The investigators in this study reviewed the adopted COVID-SCORE statements, assessed content validity, construct validity and approved changes.”*

Line 126: Why is regional representation important in this context? Please clarify.

*The sentence has been revised as follow:*

*“We aimed to have regional representativeness of academics in the panel as Indonesia is diverse across its regions.”*

Line 127: When describing the sampling technique, please use more academic/formal terms such as purposive sampling etc.

*The sentence has now been revised as follow:*

*“Thus, we started with purposively selecting sampling potential universities to represent four regions of Indonesia: Sumatera, Java, Kalimantan, and Eastern Indonesia.”*

Line 132: Did the authors follow a snowball sampling technique in the second stage of sampling? Please clarify the different stages that were used during the sampling process.

The sentence has now been revised as follow:

*“To ensure that respondents have the capacity and capability to participate in the survey, we then performed snowball sampling by asking senior lecturers to recommend a list of academics from their respective universities.”*

Line 134: Was the data collection process completed online? Please clarify this at the beginning of the methods section. Otherwise, it is confusing to the reader.

*The data collection was online and this has been mentioned in the data collection part of the method section as follow:*

*“The email contained a short description of the background and aims of the study, the process of two-cycle Delphi surveys, and a link to an online questionnaire on the Qualtrics Research Suite platform.”*

Line 137: How was this number (about 30) determined? Please clarify.

We have added the following explanation and reference to the Study Population part of the method section”

*“However, a Delphi panel usually consists of 15 to 30 participants from the same discipline, or five to 10 per category from different professional groupings [16].”*

Line 148: Please clarify why 2 languages were used in the survey instrument.

*The sentence has now been revised as follow:*

*“All statements were written in Bahasa Indonesia and English to minimize variations in interpretations among participants.”*

Line 159: Why was not the data de-identified? Why did the researcher have access to the identity of the survey participants? Please clarify.

*All experts in the panel were kept anonymous to each other but not to the researcher throughout the process. The researchers need to be able to trace responses of the experts in order to be able to link responses across the rounds*

Line 163: Please clarify - was the feedback shared with the participants?

*As stated at the end of the data collection part of the method section:*

*“The result of the second (final) cycle was analyzed, and feedback to all participants a week later.”*

Line 167: Please correct the spelling for calculated.

*This has now been corrected as follow:*

*“We calculated percentages of score for each statement.”*

Line 170: Please clarify how this decision criteria was determined. Please provide relevant references if possible.

*The following sentence and reference has been added:*

*“We used percent agreement, which is the most common definition for consensus in Delphi [17].”*

*The 75% threshold set for Moderate consensus level is derived from median threshold of previous delphi surveys as noted in the reference.*

*Ref:* *Diamond IR, Grant RC, Feldman BM, Pencharz PB, Ling SC, Moore AM, Wales PW. Defining consensus: a systematic review recommends methodologic criteria for reporting of Delphi studies. J Clin Epidemiol. 2014 Apr;67(4):401-9. doi: 10.1016/j.jclinepi.2013.12.002.*

Line 180: Please clarify what is meant by 1,5 hours.

*This has now been revised as follow:*

*“The webinar was delivered via Zoom, facilitated by the authors and completed in 90 minutes”*

Line 181: In case a respondent disagreed with the results - how was that handled by the researchers? What implications might it have for the results of the study?

*If a respondent disagreed then the results would have to be discussed with the panellists to decide the final level of agreement*

Line 189: Earlier it was mentioned that the researchers/authors had access to participant identities. This could have affected the responses from the participants. How was this handled by the authors?

*To minimize biases, we employed standard techniques that are recommended in delphi surveys, eg. Include reasons in controlled feedback; Conduct multiple rounds of surveys.*

*Ref: Hallowel MR. Techniques to Minimize Bias When Using the Delphi Method to Quantify Construction Safety and Health Risk. Construction Research Congress 2009. Published online: April 26, 2012.* [*https://doi.org/10.1061/41020(339)151*](https://doi.org/10.1061/41020(339)151)*.*

Line 287: Was this a recommendation made by the respondents?

*Yes, this recommendation was derived from respondents’ responses*

Line 325: This can be better expressed as quality of product.

*This has now been revised as follow:*

*“Thus, guidance and supporting policy are needed to attract investors to collaborate with the government and ensure public trust in products quality of products.”*

Line 337: Please correct grammatical issues with this sentence.

*This has now been revised as follow:*

*“Thus, the extent to which does public health experts have been involved in the response team remains questionable.”*

Line 344: Please provide a reference for this guideline.

*The following reference has now been added:*

*Ministry of Health. COVID 19 Infection prevention and control guideline. 5th Ed. Jakarta: Ministry of Health Republic of Indonesia; 2020.*

Line 355: Please rewrite this sentence to make it clear for the readers.

*This has now been revised as follow:*

*“However, respondents were concerned that a policy to guide the implementation of a wide range of telehealth services was unavailable”*

Line 376: Please provide references for these resources.

*The following reference has been added as requested:*

*Sukarsa, AR. Devi OS. Analysis of Indonesian Government Public Communication based on Image Restoration Theory. Jurnal Mantik. 2021;5(2):1192-1199*

Line 410: Please correct grammatical issues with this sentence.

*This has now been revised as follow:*

*“There were also issues regarding terms used for infection control, disease transmission and treatment.”*

Line 446: Please correct grammatical issues with this sentence.

*This has now been revised as follow:*

*Many non-governmental organizations and philanthropies raise funds to support those affected by the COVID-19 pandemic, including the poor, elderly, and frontline healthcare workers.”*

Line 451: Are these 2 separate departments of the government? Please clarify.

*Yes, these are separate ministries*

Line 470: Overall, the discussion section should be made more concise, as several paragraphs can be summarized into smaller ones.

*We have revised the discussion section to make it more concise*

Line 471: The results mentioned here has already been mentioned in the results section. This paragraph is thus no longer needed.

*The results have indeed been mentioned on the results section, but we believe it’s useful to revisit the key results briefly at the beginning of the discussion section to reorient the readers.*

Line 478: The results mentioned here has already been mentioned in the results section. This paragraph is thus no longer needed.

*The results have indeed been mentioned on the results section, but we believe it’s useful to revisit the key results briefly at the beginning of the discussion section to reorient the readers.*

Line 513: Please summarize the discussion on communication in a more concise way.

*The discussion on communication has now been revised to be more concise*

*Line 612: Please clarify the meaning of higher levels of evidence.*

*This refers to the notion of hierarchy of evidence, in such context this could encompass, e.g. Descriptive surveys, qualitative studies, systematic review, metasynthesis, etc*

Reviewer #5: Thanks for the authors for introducing us how medical and health academics view the Indonesian government's handling of the COVID-19 and which areas of health systems need to be prioritized. However, there are several concerns:

1. Although the author explains the reasons for using the Delphi method in page 5 lines 93-95, this kind of research does not require the Delphi method (time-consuming and laborious). The same result would have been obtained if a questionnaire had been used. The commonly used Delphi method is only used to forecast, put forward the index system and determine the specific index.

*The delphi technique is commonly used in developing consensual guidance, not necessarily used to forecast. A traditional survey would have been difficult to conduct during the pandemic. An online survey not using the delphi method would require much more respondents and would get low response due to less engagement*

2. In the research design section, the COVID-19 assessment scorecard consists of 19 policy statements [1], but the author noted 20 in the manuscript.

*The research design section as follow mention 20 statements:*

*“The COVID-SCORE consists of twenty policy statements about improving public health communication and health literacy, facilitating robust surveillance and reporting, developing pandemic preparedness, strengthening the health system, ensuring the health and social equity, and ensuring comprehensive confinement and de-confinement strategies. The twenty statements from COVID-SCORE were then modified into three types of questionnaires: Likert scale survey, short comment, and ranking scale.”*

3. It is odd for the authors to use the Cambridge Dictionary's definition of scholarship to select a study population. The views of the health sector and healthcare providers on policies to control COVID-19 are even more important. The study was underrepresented.

*The Delphi method is primarily used to elicit opinions of experts, thus the scholarship definition is highly relevant*

4. In the results section, it is more intuitive to use the expert status table. For example, age can be expressed in three groups.

*We feel that the descriptive characterization of the experts is sufficient*

5. Please explain in the methods section why the P value is calculated. In general, the Delphi method does not require the calculation of P- values.

*This has been explained in the method section as follow:*

*“We calculated p-values to assess the significance of the change in the degree of consensus for each statement between the cycles”*

6. It is too tedious to present all policy statements in the results. Simplify them.

*We believe it is important to present all policy statements in the results*

7. From the results of this study, moderate consensus was reached on only 5 statements, low consensus was reached on 13 statements, and no consensus was reached on 2 statements. In the Delphi method, the reliability of the results should be proved. However, the expert' authority coefficient (Cr) and the coefficient of variation (CV) are necessary. The reliability and representativeness of the manuscript are worrisome.

*We follow the Guidance on Conducting and Reporting DElphi Studies (CREDES). This guidance does not call for calculating and reporting of expert' authority coefficient (Cr) and the coefficient of variation (CV)*

Reviewer #6: The authors present an important application of expert elicitation to guide policy decision making in the context of COVID-19. The methods for the expert elicitation are clearly reported, and the results are presented in a well-organized manner. The authors have responded satisfactorily to the comments from both rounds of reviewers. I have a couple of minor comments.

First, pg 15, please specify in text that no statements received a high consensus level.

*This has now been incorporated in the second paragraph of the results section as follow:*

*“Out of 20 survey statements, we achieved a moderate consensus level for five statements, 13 low consensus levels, and no consensus for two statements. No statements received a high consensus level.”*

Second, line 318, the statement “the government should involve domestic private manufacturers” is phrased differently from all other scorecard descriptions which state whether the experts think the government has done the task. Could the authors please clarify if this should in fact be past tense (has) or if future tense (should) is correct. If the latter, then please clarify why the interpretation for this statement is different.

*This has now been revised as:*

*“**Respondents generally agreed that the government can require private manufacturers to produce critical equipment rapidly. The government has involved domestic private manufacturers to produce critical equipment rapidly”*

Finally, I saw no mentions to the supplemental materials made in text. Please add references to these materials in the requisite sections. Additionally, the code book submitted in appendix 3 appears to be incomplete with descriptions absent and titles that do not correspond to the data elements in the data file. Have the authors considered attaching the complete survey as an additional supplement?

*We’ve added the following sentence to the data collection part of the method section:*

*“**The survey questionnaire is available from the authors on request”*

*We’ve also added the following sentence to the analysis part of the method section*

*“The dataset and code book has been made available as supplementary files to this manuscript.”*
